# Sentinel lymph node mapping: current applications and future perspectives in thyroid carcinoma

**DOI:** 10.3389/fmed.2023.1231566

**Published:** 2023-10-24

**Authors:** Isabella Merante Boschin, Loris Bertazza, Carla Scaroni, Caterina Mian, Maria Rosa Pelizzo

**Affiliations:** ^1^UOC Endocrinology, Dipartimento di Scienze Chirurgiche Oncologiche e Gastroenterologiche (DiSCOG), Università degli Studi di Padova, Azienda Ospedale-Università di Padova, Padua, Italy; ^2^UOC Endocrinology, Dipartimento di Medicina (DIMED), Università degli Studi di Padova, Azienda Ospedale-Università di Padova, Padua, Italy; ^3^Dipartimento di Scienze Chirurgiche Oncologiche e Gastroenterologiche (DiSCOG), Università degli Studi di Padova, Padua, Italy

**Keywords:** sentinel lymph node, thyroid cancer, papillary thyroid carcinoma, differentiated thyroid carcinomas, lymphadenectomy

## Abstract

Sentinel lymph node (SLN) mapping is a standard, minimally-invasive diagnostic method in the surgical treatment of many solid tumors, as for example melanoma and breast cancer, for detecting the presence of regional nodal metastases. A negative SLN accurately indicates the absence of metastases in the other regional lymph nodes (LN), thus avoiding unnecessary lymph nodal dissection. Papillary thyroid carcinoma (PTC) is the most common type of thyroid carcinoma (TC) with cervical LN metastases at diagnosis in 20–90%, and nodal involvement correlates with local persistence/recurrence. The SLN in PTC is an intraoperative method for staging preoperative N0 patients and for detecting metastatic LNs “in and outside” the cervical LN central compartment; it represents an alternative method to prophylactic central neck node dissection. In this review we summarize different methods and results of the use of SLN in TC. The SLN identification techniques currently used include the selective vital-dye (VD) method, 99mTc-nanocolloid planar lymphoscintigraphy with intraoperative use of a hand-held gamma probe (LS), the combination LS + VD, and the combination LS and preoperative SPECT–CT (LS + SPECT/CT). The application of the SLN procedure in TC has been described in many studies, however, the techniques are heterogeneous, and the role of SLN in TC, with indications, results, advantages and limits, is still debated.

## 1. The concept of the sentinel lymph node

The term “lymphatic” was coined by Thomas Bartholin in 1653, since then many anatomists and physicians have committed to the study of this elusive system (1, 2). Its role in tumor spread has been researched in order to understand the complicated mechanism of tumor metastasis. The sentinel lymph node (SLN) may be considered a milestone in the pathway for defining the development of lymphatic metastasis (1, 2).

The anatomist J.H. Gray, in the 1930s, performed an analysis of the lymphatic system utilizing colloidal thorium dioxide injected into surgical wounds and observed the lymphatic pathways that formed anastomoses at capillary or lymphatic trunk by which some of the lymph could be carried into different regional lymph nodes. The author concluded that the regional lymph nodes draining a primary tumor will be the first and most likely site of node metastases (3, 4).

In the 1950s, Weinberg began using blue dye during gastric and lung carcinoma operations with the specific goal of highlighting “the primary nodes” draining from the tumor, on the assumption of giving them a blue color by making them easily observable (5, 6). A few minutes after injection, blue dye stained the lymph vessels and the lymph nodes: the lymph nodes mapping with blue dye had the direct consequence of avoiding unnecessary node dissections (5, 6).

Later, a radiopaque contrast agent was introduced and subsequently the two techniques – blue dye and radiopaque contrast – were used in combination (4). From the 1960s, attention turned to the lymph nodes of testicular and penile neoplasms in which the lymphatic fluid passed through a tight site. Lymphography of the testicle or penis helped to clarify the concept of selective drainage from tumors in these organs by highlighting that lymphatic drainage from one area goes to a specific group of lymph nodes (4, 7, 8). In Halsell et al. (9) processed lymphograms of the breast injecting directly into the lymphatics. In 1969, at the Universida Nacional de Asuncion Facultad de Ciencias Medicas in Asuncion, Paraguay, R.M. Cabañas graduated with a thesis on the lymphatic drainage and the lymph nodes in a series of primary cancers using lymphography: the work required the performance of 250 lymphograms of various tumors, including penile, testicular, breast, melanoma, anal and rectal, and lymphomas, between 1965 and 1968 (4).

In Cabañas (10) presented an extension of his study on penile carcinoma to the Society of Surgical Oncology in New York City. The aim of his paper was, first, to emphasize the evidence of a specific lymph nodes group, the so-called “sentinel lymph node” (SLN), in the lymphatic system of the penis, second, to stress the importance of routine, bilateral dissection of the SLN in the surgical treatment of penile carcinoma, and third, to illustrate and explain the SLN technique. He concluded that SLN status could be used for defining the indication for a complete lymph node dissection.

Morton et al. (11) published the first clinical report documenting the results of SLN surgery in 223 melanoma patients with preoperative negative regional lymph nodes using isosulphan blue dye intraoperatively to make lymphatic drainage evident from the primary cutaneous melanoma to the SLN. The investigators supported their first hypothesis that a limited number of lymph nodes receiving drainage from cutaneous injection site of blue dye would result in blue staining (11).

At the “1st International Congress on the Sentinel Node in Diagnosis and Treatment of Cancer” in 1999, the SLN was defined as the first lymph node draining the lymphatic flow from a primary cancer (12, 13). Subsequently, the SLN has rapidly established as a diagnostic method for many solid tumors, such as melanoma, vulvar carcinoma, penile cancer, colorectal cancer and breast cancer. Many following studies have investigated the application of SLN in other cancer types, including thyroid carcinoma (14–16).

Three conditions are required for satisfying the concept of SLN: 1) the presence of an ordered and expected pathway of lymphatic drainage from the tumor site to the regional lymph nodes, 2) the sequential progression of tumor cells moving through the lymphatics to a primary lymph nodes group, and 3) the SLN filtration effect on the afferent lymph flow trapping the tumor cells (1).

The SLN is a minimally-invasive diagnostic technique carefully detecting the presence of regional lymph node metastases: a negative SLN predicts accurately the absence of node metastases in the other regional nodes, thus avoiding unnecessary regional node dissection (1, 17).

The SLN in thyroid carcinoma (TC) was first reported in 1998 by Kelemen et al. (18) in a study on SLN detection in 17 patients affected by TC using 1% Patent Blue V dye: the detection rate was 88%, and the SLN was positive for metastases in 42% of patients. Subsequently, numerous studies were carried out to evaluate different techniques and procedures for SLN detection.

## 2. Thyroid carcinoma

Thyroid nodules are frequent in the general population, with a prevalence as low as 4–5% at clinical examination and up to 50–67% at ultrasonography and autopsy studies (19, 20). Thyroid nodules are malignant in about 5%, colloid nodules, cysts and thyroiditis nodes in about 80%, and benign follicular neoplasms in 10–15% (19–21).

Thyroid carcinoma (TC) is the most common endocrine cancer. TC is reported as the 11th most frequent cancer of all cancers, although it is the fifth most common cancer by incidence in females with a global incidence rate three to four times that in men. The highest TC incidences have been observed in North America, Australia, New Zealand, East Asia and Southern Europe (22–26).

Histologically, in the thyroid gland there are two main cell types, follicular cells and the C or parafollicular cells. The differentiated thyroid cancers (DTC), i.e., papillary thyroid cancer (PTC), follicular thyroid cancer (FTC) and anaplastic thyroid cancer (ATC), and to poorly differentiated thyroid cancers derive from the follicular cells, the medullary thyroid carcinoma (MTC) from the C cells or parafollicular cells (27–29).

Over 90% of TC cases are well differentiated DTCs, mainly PTC (85% of DTCs) and FTC (25, 26). Exposure of the thyroid to radiation in childhood, age, female gender, family history and Hashimoto’s thyroiditis are risk factors for the incidence of DTC. The age at radiation exposure in the development of TC is also important: in the first decade after the Chernobyl accident, in some regions of Belarus, a 100-fold rising in TC was observed in the population below the age of 15 at the time of exposure (23, 25, 30). Epidemiological studies have demonstrated the first-degree relatives of subjects with this neoplasia, have a four- to ten-fold increased risk of DTC (27, 28).

PTC is classically characterized by its papillary appearance and distinctive nuclear signs, and more than 10 histological variants have been documented. It is considered an indolent tumor with a 30-year survival rate of over 90% (30, 31). PTC is a lymphophilic cancer and cervical lymph node (LN) metastases are frequent, increasing probability of loco-regional persistence/recurrence of the disease: tumor size, extracapsular invasion and multifocality are factors associated with LN metastases (32, 33). Distant metastases are a strong predictor of a poor prognosis with a reported death secondary to the thyroid cancer in 43–90%; hematogenous distant metastatic disease is rare, the authors report 1 to 2% of PTC cases with metastases outside the neck or mediastinum at the time of diagnosis, in particular to the lung and bone (28, 32, 33).

FTC represents about 10–15% of DTCs, typically diagnosed at histology rather than at cytology as with PTC. FTC is more aggressive in contrast to the overall indolent behavior of the other classical well DTCs. The distant metastatic disease, mainly located in the lungs and bone, is reported in 3 to 30% of cases, while LN metastases are rare (34–36).

## 3. Lymph node metastases in papillary thyroid carcinoma

The intra and extra thyroid lymphatic drainage is a complex network. In PTC, the central neck compartment is the most frequently involved in metastatic disease, although node metastases in lateral and mediastinal compartments are also frequent (37–39). The lymphatic channels of the thyroid capsule cross-communicate with the isthmus and the opposite lobe, consequently the direction of the lymphatic fluid draining by intra-thyroid capillaries is not always predictable (40, 41).

The isthmus and the medial superior portion of the thyroid lobes are usually drained by the superior lymphatic vessels, rising in front of the larynx and reaching subdigastric LN of the internal jugular chain; the media inferior lymphatics terminate in the pretracheal lymph nodes; the lateral lymphatics drain to the superior LN of the internal jugular vein. The location and the anatomic borders of LN groups in the neck have been described by many different authors, the American Joint Committee on Cancer (AJCC) and the American Society of Head and Neck Surgery (AHNS) classifications are the most commonly used (42–45).

Between 15 and 50% of PTCs have cervical LN metastases at diagnosis, but microscopic metastases have been found in up to 80% (16, 46). The different extent of the neck node dissection determines the different prevalence of regional lymphatic metastases reported by various authors (39–41).

Metastases occur in the central compartment (VI level), including the pretracheal, paratracheal, perithyroidal and precricoid nodes, in approximately 90% of cases (metastases in the Delphian lymph node are associated with latero-cervical metastases in up to 33% of cases) (45–48). The lateral compartment is involved in between 51 and 100% of cases in different series, in particular the lower jugular compartment (IV level) is the second most commonly affected site, following by the middle (III level) and upper jugular compartments (II level) (46–52). Less common are V- and VII-level metastases (from 2 to 15%). The contralateral LN is involved in up to 18–25% of PTC cases (45–47), while lateral skip metastases are found in up to 20% (17, 52, 53).

In PTCs smaller than 10 mm, the LN metastases are rare and are generally found in the central compartment (40, 53). They are an unfavorable prognostic factor, in particular for disease-free survival, rather than for overall survival, while the other principal adverse prognostic factors are male gender, age 55 years or older, tumor size more than 3 cm and extra-nodal extension (37).

## 4. Neck node dissection in the management of PTC

The thyroid lymphatic drainage network is quite complicated that in thyroid surgery the direction of the tumor lymphatic drainage is not always predictable (27–29, 53).

Neck cervical LN dissection is based on preoperative staging, currently requiring both total thyroidectomy and node dissection in cases with positive LN. The management of preoperative N0 status is a controversial issue; given the high rate (up to 80%) of occult micro-metastases and the resulting higher rate of locoregional persistence/recurrence in PTC, treatment can range from “node picking” to ipsilateral or radical central node dissection (CND) (39–41, 53, 54).

Neck CND exposes the patient to a higher risk of damaged recurrent laryngeal nerves (RLN) and higher rates of hypoparathyroidism, leading to over-treatment in patients with negative LNs. Although many argue that the risks of CND are no greater than total thyroidectomy, especially when carried out by an experienced surgeon, there is much debate about this (17, 54–56).

In a review of seventeen studies involving 1,929 patients published in 2009, after CND, transient hypocalcaemia is reported in 3.6–60.0%, permanent hypocalcaemia in 0.0–14.4%, temporary RLN injury in 0.0–25.0%, and permanent RLN injury in 0.0–11.5% (57).

The standard ATA guidelines recommend therapeutic CND only for patients with preoperative central LN metastases. These guidelines also recommend prophylactic CND (ipsilateral or bilateral) in patients without preoperative central LN metastases (cN0) but with advanced primary tumors (T3 or T4) or preoperative lateral LN metastases (cN1b), or if the LN histological status information is required to plan further steps in therapy. According to the ATA guidelines the prophylactic CND is not recommended in preoperatively cN0 patients with T1 and T2 tumors (58).

PTC patients are classified cN0 on the basis of clinical examination and high-resolution neck ultrasonography (hrUS). However, neck hrUS may yield false-negative results in up to 30% of patients due to an inability to detect occult LN metastases (58, 59).

## 5. SLN procedures in TC

The SLN procedure in PTC is an intraoperative method of staging for metastatic LNs “in and outside” the central compartment in cN0 patients, and for identifying patients who might benefit from lymph node dissection instead of prophylactic CND. The SLN is the first regional lymph node or nodes group affected by metastases from a primary tumor, a negative SLN accurately predicts the lack of metastases in the other LNs (60).

The accurate diagnosis of LN metastases in PTC is recognized as crucial and significantly affects treatment and survival (40, 41).

The following SLN identification techniques are currently used: the selective vital-dye (VD) method, 99mTc-nanocolloid planar lymphoscintigraphy with intraoperative use of a hand-held gamma probe (LS), a combination of the LS and VD methods (LS + VD), 99Tc- nanocolloid planar lymphoscintigraphy with preoperative SPECT–CT and intraoperative use of a hand-held gamma probe (LS-SPECT/CT) (61).

The most frequently used blue dyes are isosulphan blue, patent blue violet (V), sodium blue and methylene blue. The literature search of Pubmed we conducted yielded 33 papers on SLN in TC using the blue dye method, specifically 6 using isosulphan blue, 8 using patent blue V, and 19 using methylene blue. Focussing on those papers on SLN detection with the highest number of patients, in 2010 Cunningham et al. examined 211 PTC patients using 1% isosulphan blue with a detection rate (DR) of 91%; in 2006 Rubello et al. used patent blue V in 153 PTC cases with a DR of 69%; in 2020 I. Markovic et al. used methylene blue in 153 PTC patients with a DR of 91.8% (62–64). Using methylene blue in 20 MTC patients, in Santrac et al. (65) achieved a DR of 100%, with 10% positive for metastasis. In Wang et al. (66) described the use of indocyanine green (ICG) with methylene blue for detecting SLN in 45 micro-PTC patients with a longer operative time but a lower rate of hypoparathyroidism: the authors concluded that for SLN in TC ICG in combination with methylene blue is feasible, safe and also clinically significant for protection of the parathyroids. In Moskalenko et al. (24) evaluated the effectiveness and safety of intraoperative SLN detection in 187 patients using for the first time a 1% toluidine blue aqueous solution, and with a DR of 97.6% they found it to be no less accurate that other blue dyes.

With the VD method at surgery, the vital blue dye intra- or peri-tumoral injection is performed generally utilizing a tuberculin syringe (24, 62, 64–67). The authors recommend not mobilizing the thyroid before injection to ensure intact lymphatic drainage. The blue dye can be usually observed moving through lymphatics to the SLN within seconds, sometimes only after 1–2 min. The blue-colored lymph nodes are excised very cautiously to avoid removing the parathyroid glands, which can also be accidentally colored blue. After dissection, the SLNs are submitted to histopathology for frozen section analysis. The main limits of the VD procedure include: 1) the potential disruption of the lymphatics draining the tumor, 2) the difficult identification of the SLNs located outside the central compartment, 3) the risk of removing blue stained parathyroid glands, and 4) the procedure is laborious requiring experience (24, 62, 64–69).

The literature search of Pubmed yielded 14 papers on SLN in TC using the LS method. In Carcoforo et al. (70) assessed 345 PTC patients and detected SLNs in 100% of them using the LS technique, with 22.6% positive for metastases. In Kim et al. (71) evaluated 16 MTC patients with the LS method and detected SLNs by radioisotope in 87.5%. After the US-guided peri- or intra-tumoral injection of 99mTc-albumin nanocolloid particles the lymphatics from the injection site are visualized. The skin projection of the SLN can be validated by external counting with a hand-held collimated gamma probe and scored with a permanent marker (70–77).

After a varying time interval (2–24 h) the patient is taken to the operating room and, following thyroidectomy for avoiding interference from radioactivity of the primary tumor, the central and the lateral node compartments (through the incision and the skin surface respectively) are scanned with a hand-held collimated gamma probe for radioactive lymph nodes. The authors reported SLN detection being feasible up to 24 h post injection. The SLN is selectively removed, the radioactivity of the lymphatic bed is monitored with the hand-held gamma probe to verify completeness of SLN dissection and the SLN is sent to the histopathology for searching for metastasis. The use of preoperative lymphoscintigraphy with radiocolloids and intraoperative gamma probe detection, first described by Rettenbacher et al. (73), was introduced as a SLN technique in TC to overcome some of the drawbacks of the VD technique. Lymphoscintigraphy is a remarkable technique for visualizing the lymphatic vessels and the SLN. According to some authors, it offers important advantages over the VD method: 1) the preoperative US-guided injection of radioactive particles removes the risk of lymphatic vessels damage at surgery, 2) the SLN can be localized in and outside the central compartment, and 3) there is no radioactive particles capture by the parathyroids (70–77).

In the LS + VD procedure, the SLN is localized through a combination of preoperative lymphoscintigraphy, intraoperative hand-held gamma probe and blue-dyes. The LS + VD method was first described in 6 PTC patients by Catarci et al. (74). Two hours prior to surgery they administered an intra-tumoral injection of 99mTc-labeled colloidal albumin to visualize the SLN at lymphoscintigraphy. At surgery, Patent Blue V (2.5%) was intratumorally injected: the blue stained SLN was subsequently localized by a hand-held gamma probe. The SLN was identified in all cases, and the authors concluded that these techniques have a complementary role (74). Subsequently, other authors have evaluated this combination technique, for example, Lee et al. (78), Huang et al. (79), Assadi et al. (80), and Gelmini et al. (81).

With the LS-SPECT/CT technique, the SLN is localized by the combination of the 99mTc-nanocolloid planar lymphoscintigraphy with the preoperative SPECT/CT imaging and intraoperative use of a hand-held gamma probe (82–85).

Recently, nanotechnology has developed, and nanocarbons (CN) have been largely used as a LN tracer in malignancies. In the CN suspension, the nanosized carbon particles have an average diameter of 150 nm: the CN particles are peritumorally injected and rapidly undergo phagocytosis by macrophages, subsequently enter the lymphatics and accumulate in the LNs staining them black (86). This procedure has been applied in the detection of SLNs in breast and gastric cancers. In 2012, a study evaluated 100 micro-PTC patients who were given a peritumorally injection of CN suspension; within a few minutes the limphatic flow and the black-stained SLN in the central compartment were individualized. The DR with the CN method was 93.3%, with 61.1% positive for SLN metastases (87). In Zhang et al. (88) examined the feasibility and efficacy of Indocyanine green (ICG) and CN intrathyroid injection to localize the SLN in 40 micro-PTC patients.

Recently, new techniques have been proposed for detecting SLN in TC, such as 68Ga-tilmanocept PET/CT. In de Vries et al. (89) proposed a study of SLN in TC according to a clinical protocol using 68Ga-tilmanocept PET-CT and ICG-99MTc-nanocolloid in ten patients with DTC and MTC. The patients will be given a sequential US-guided intra- or peritumoural injection of 68Ga-tilmanocept and ICG-99mTc-nanocolloid, followed 15–60 min later by 68Ga-tilmanocept PET/CT. The next day the site of the SLN will be preoperatively defined using a hand-held gamma probe and marked on the skin. At surgery, the SLN will be excised using the 68Ga-tilmanocept PET/CT images, the skin markings, a hand-held gamma probe and a fluorescence camera. According to the authors, the promising new imaging modality of 68Ga-tilmanocept PET-CT reduces the shine-through effect described in LS method, by better localizing the SLN close to the tumor (89).

## 6. Application of SLN in TC

In order to evaluate the “state of the art” of the SLN method in TC, in 2016 our surgical team looked at 41 studies on SLN detection in TC: 26 using the VD technique (patient numbers ranging from 9 to 300), 12 using the LS technique (1 to 374 patients) and 3 using the combination LS + VD (6 to 45 patients) (90). The SLN was successfully visualized in 0–100%, 64–100% and 98–100% of cases, respectively, and was positive for metastases in 14–86%, 16–100% and 50–67% of cases, respectively (90). The authors concluded that the SLN technique for PTC represented a rigorous method for identifying node metastases more readily than preoperative clinical and hrUS examination, and that therefore a randomized clinical trial assessing the SLN efficacy and usefulness on management and long-term patient outcomes was justified.

From our literature search, until today, we identified 4 meta- analyzes of SLN techniques in TC.

In Raijmakers et al. (91) realized the first meta-analysis considering a total of 457 patients in 14 studies carried out between 1998 and 2007 and evaluating the detection rates of different SLN techniques (blue dye or radiotracer) in PTC. Ten of these studies (329 patients) used the blue dye technique with a pooled SLN detection rate of 83%, four studies (128 patients) used 99mTc-colloid with a pooled SLN detection rate of 96%. There was a false-negative rate (FNR) of 11.3% in one of the studies utilizing 99mTc-colloid (91).

In Balasumabrian et al. (92) carried out a second meta-analysis and review of SLN in TC. They looked at 23 studies with a total of 1,011 patients: 17 (832 patients) using the vital-dye technique (VD), 4 (129 patients) using the radioisotope technique with lymphoscintigraphy (LS), and 2 (50 patients) using the combination LS + VD. The overall SLN DRs were 83.7% for VD, 98.4% for LS and 96% for LS + VD. The overall FNRs were 7.7% (0–33%) for VD, 16% for LS and 0% for LS + VD. The authors concluded that the results of these numerous studies provide evidence to support the opportunity for a multicentre, randomized, controlled trial and a more rigorous evaluation of the SLN technique in TC; at present, the role of routine prophylactic CND in PTC is still debated, and this is an impetus for further research in this area (92).

In Kaczka et al. (93) carried out a third meta-analysis of the SLN technique in TC which took in 25 studies with a total of 1,100 patients: 891 patients in 18 studies using the VD technique, 160 patients in 4 studies using the LS technique and 49 patients in 2 studies using the combination LS + VD. The SLN was detected in 83.1, 98.8 and 98.8% of cases, respectively (93).

In the last meta-analysis, carried out in 2019, Garau et al. (61) drew on as many as 47 studies comprising a total of 2,498 patients: 1438 patients in 27 studies using VD, 782 patients in 11 studies using LS, 161 patients in 5 studies using the combination LS + VD and 117 patients in 4 studies using LS and preoperative SPECT/CT (LS-SPECT/CT). The overall SLN detection rates were 83% for VD (77–88%), 96% for LS (90–98%), 87% for LS + VD (65–96%) and 93% for LS-SPECT/CT (86–97%). The authors concluded that 99mTc-nanocolloids offers a higher SLN detection rate than the VD procedure, and the identification of the SLN outside the central neck compartment is improved by the contribution of the preoperative SPECT/CT.

In Garau et al. (37) carried out a literature search of meta-analyzes and systematic reviews of SLN in TC and found that evidence-based data on the SLN in PTC were increasing. However, they concluded that a consensus on SLN technique is required by the nuclear medicine community and that SLN identification, removal and analysis needed to be standardized.

Subsequent to these meta-analyzes numerous other papers have followed over the years.

In Wang et al. (66) evaluated indocyanine green (ICG) combined with methylene blue for detection of SLN in 90 microPTC comparing 45 patients with ICG and methylene blue (group A) versus 45 patients with only methylene blue (group B). The sensitivity and accuracy rates were higher in group A but the operating time was longer; the combination of ICG and methylene blue resulted significant to protect the parathyroid glands and the rate of hypoparathyroidism was lower in group A. In Kim et al. (71) evaluated the utility of lateral neck SLN biopsy in detecting lateral neck node dissection in patients affected by MTC. In Rodriguez et al. (94) published a study on the detection of SLN in 55 PTCs using LS procedure: the SLN detection, the sensitivity and the specificity were 96.36, 86.21 and 100%, respectively. The authors concluded that the results of sensitivity and specificity of SLN procedure are similar in literature even if the variability of the different describing methods limits their interpretation. In Yan et al. (23) carried out a prospective study on SLN technique in the lateral neck compartment and prophylactic lateral neck node dissection in 78 PTCs. The detection rate, sensitivity, specificity and accuracy rate of SLN in the lateral neck compartment were 98.7, 87.1, 98.7 and 93.6%, respectively. The authors concluded that SLN can be used as an indicator for identifying PTCs who may benefit from lateral neck node dissection and avoiding prophylactic lateral neck node dissection. In Wang et al. (95) realized a study on the tumor targeting peptide TMVP1 – modified polymer nanomaterials loaded with the near-infrared (NIR) fluorescent dye indocyanine green (ICG) for preparing a molecular imaging TMVP1-ICG nanoparticles (NPs) to identify tumor metastases in SLN imaging. *In vitro* cell and *in vivo* mouse experiments evidenced that TMVP1-ICG-NPs have a good targeting capability to tumors *in situ* and to metastatic SLN by binding to vascular endothelial growth factor receptor-3 (VEGFR-3) and subsequently were used to perform imaging-guided photothermal therapy (PTT). The authors concluded that TMVP1-ICG-NPs can be proposed as a promising theranostic nanomedicine and a new strategy for providing real-time NIR fluorescence imaging and intraoperative PTT for patients with metastatic SLN (95).

## 7. Future perspectives

The role of SLN method in TC, with indications, advantages and drawbacks, has been controversially debated. SLN in PTC is proposed as an intraoperative method for staging preoperative N0 PTC, and selecting patients to undergo a CND beneficially and optimized radio iodine ablation treatment.

The LS technique has a higher detection rate than VD, while the combination of LS and SPECT/CT has been demonstrated to be the superior method for providing clearer images and for identifying SLNs outside the central compartment.

Some authors consider LS to offer important advantages over the VD method such as no risks of lymphatics damage at surgery by the preoperative US-guided injection of radioactive particles, the possibility to visualize the SLN outside the central compartment, no risk of radioactive particles capture by the parathyroid glands. In the LS method thyroidectomy should be performed before SLN localization and dissection, because of the so called “shine-through” effect: the SLN is selectively removed and the lymphatic bed is scored with a hand-held gamma probe to check for radioactivity reduction (4, 90). The combined LS-SPECT/CT technique represents an enhancement of the LS method (82–85).

The principal limitations of the LS technique are as follows: 1) the intraoperative radioisotope method requires access to a Nuclear Medicine Unit and/or the attendance of a nuclear physician at the time of surgery, 2) radio-protection is required in the operating room, and 3) a dedicated, experienced anatomic pathologist is required at surgery to detect lymphatic metastases on extemporaneous histologic examination (37, 90). Rapid thyroglobulin (TG) assay in fine needle aspiration (FNA) of SLNs may be an alternative method for detecting metastases.

Positive SLN in other cancers is strategical in planning surgery and subsequent therapies. In the application of SLN method in TC, on the one hand, it is necessary to achieve a consensus on the techniques, and on the other hand, there is a need for controlled, randomized and long-term clinical trials to investigate the value of metastatic SLN in persistence and recurrence of disease for better planning node dissection and the postoperative radioactive iodine therapy.

## 8. Conclusion

The SLN is the first lymph node(s) receiving lymphatic drainage from a specific primary cancer, it is the first node (s) involved with metastases and it reflects the metastatic status of the others draining nodes (86). The SLN procedure is a surgical lymphatic mapping method for identifying node metastases in preoperative No carcinoma. The SLN in TC was first reported in 1998 and since then many studies have been performed on this question. However, the value of the SLN in terms of indications, benefits and limitations is still under discussion (90, 91).

SLN detection in TC is safe and feasible. The LS technique has a higher detection rate than VD, and the combination with SPECT/CT has been shown to be superior to conventional planar lympho scintigraphy in providing more accurate images, in localizing SLN in and outside the central compartment and consequently better planning for surgery.

The SLN procedure is little used in TC as it has no recognized strategic role in the therapeutic plans of other more aggressive carcinomas; it is also not common for mainly logistical reasons. Furthermore, thyroid surgery can also be carried out often without preoperative diagnosis and staging in low-volume centers, which have neither a Nuclear Medicine unit equipped to perform SLN scintigraphy, nor a Pathology Anatomy unit with a dedicated pathologist with experience in extemporaneous analysis.

A further factor restricting the use of SLN is the fact that TC prognosis is based on overall survival rate, which is generally favorable even with persistent and recurrent disease (90% after 30 years), rather than on disease-free survival or especially on early postoperative restaging, necessary for evaluating the radicality of surgery. Hence, no distinction is generally made between persistence and recurrence. With this in mind, we propose to validate the SLN scintigraphy procedure through a prospective study using dynamic post-surgical re-staging (96) based on early (after 2 months) postoperative values of basal (and possibly stimulated) TG combined with negative anti-TG antibodies and ultrasonography without the directly use of complementary therapy with 131I.

The primary purpose of SLN is to make thyroid surgery safer, wherever it is performed, by avoiding accompanying total thyroidectomy with unnecessary prophylactic level VI lymph node dissection, which even in experienced hands is not without its complications, but also by avoiding the use of complementary therapies, thereby preparing the ground for more conservative choices for low-risk PTC. Early detection instead of dosable TG to indicate disease persistence would also help to dispel the myth that TC has a high recurrence rate, which implies a previous recovery. A controlled, randomized clinical trial evaluating the efficacy of SLN technique in TC is needed.

## Author contributions

IB carried out the literature searches and wrote the paper. MP contributed to the drafting of the manuscript. LB contributed in drafting the manuscript and edited the bibliography. CS and CM reviewed the final version of the manuscript. All authors contributed to the article and approved the submitted version.
